# Lipopolysaccharide Induces Mitochondrial Fragmentation and Energetic Shift in Reactive Microglia: Evidence for a Cell-Autonomous Program of Metabolic Plasticity

**DOI:** 10.3390/toxins17060293

**Published:** 2025-06-09

**Authors:** Marcelle Pereira dos Santos, Vitor Emanuel Leocadio, Lívia de Sá Hayashide, Mariana Marques, Clara Fernandes Carvalho, Antonio Galina, Luan Pereira Diniz

**Affiliations:** 1 Instituto de Ciências Biomédicas, Universidade Federal do Rio de Janeiro, Rio de Janeiro 21941-902, RJ, Brazil; marcellebenac@gmail.com (M.P.d.S.); vitoremanuelarleo@gmail.com (V.E.L.); livia_hayashide@id.uff.br (L.d.S.H.); marianamarques002@gmail.com (M.M.); 2 Instituto de Bioquímica Médica Leopoldo de Meis, Universidade Federal do Rio de Janeiro, Rio de Janeiro 21941-902, RJ, Brazil; clara.carvalho@bioqmed.ufrj.br (C.F.C.); galina@bioqmed.ufrj.br (A.G.)

**Keywords:** microglia, BV-2 Cell Line, LPS, neuroinflammation, mitochondrial fragmentation, energetic failure, metabolic dysfunction, reactive oxygen species, nitric oxide, mitophagy, central nervous system

## Abstract

Microglia, the resident immune cells of the central nervous system (CNS), play essential roles in maintaining brain homeostasis. While transient activation is protective, chronic microglial reactivity contributes to neuroinflammatory damage and neurodegeneration. The mitochondrial mechanisms underlying this shift remain poorly understood. Here, we investigated whether lipopolysaccharide (LPS) induces coordinated mitochondrial and metabolic alterations in BV-2 microglial cells. LPS stimulation (100 ng/mL, 24 h) induced a reactive phenotype, with increased Iba1 (+82%), F4/80 (+132%), and Cd68 (+44%), alongside elevated hydrogen peroxide (~6-fold) and nitrite (~45-fold). Cytotoxicity increased by 40% (LDH assay), and cell viability dropped to ~80% of the control (MTT). Extracellular lactate increased, indicating glycolytic reprogramming. However, LPS-primed cells showed greater ATP depletion under antimycin A challenge, reflecting impaired metabolic flexibility. Hoechst staining revealed a ~4-fold increase in pyknotic nuclei, indicating apoptosis. Mitochondrial dysfunction was confirmed by a 30–40% reduction in membrane potential (TMRE, JC-1), a ~30% loss of Tomm20, and changes in dynamics: phospho-Drp1 increased (+23%), while Mfn1/2 decreased (33%). Despite a ~70% rise in Lamp2 signal, Tomm20–Lamp2 colocalization decreased, suggesting impaired mitophagy. High-resolution respirometry revealed decreased basal (−22%), ATP-linked (24%), and spare respiratory capacity (41%), with increased non-mitochondrial oxygen consumption. These findings demonstrate that LPS induces mitochondrial dysfunction, loss of metabolic adaptability, and increased apoptotic susceptibility in microglia. Mitochondrial quality control and energy flexibility emerge as relevant targets to better understand and potentially modulate microglial responses in neuroinflammatory and neurodegenerative conditions.

## 1. Introduction

Microglia are the resident immune cells of the central nervous system (CNS) and play a central role in maintaining brain homeostasis. Under physiological conditions, they continuously monitor the neural environment, eliminate cellular debris, remove excess synapses, and respond to pathogens via phagocytosis [1]. In addition to their scavenging functions, microglia secrete trophic factors that support neuronal survival, modulate synaptic plasticity, and guide the proper formation of neural circuits during development.

Upon injury, infection, or in the context of neurodegeneration, microglia transition from a surveillant to a reactive phenotype, characterized by morphological remodeling, increased expression of surface markers such as ionized calcium-binding adaptor molecule 1 (IBA1), cluster of differentiation 68 (Cd68), and F4/80 glycoprotein (F4/80), and the release of pro-inflammatory cytokines, chemokines, reactive oxygen species (ROS), and nitric oxide (NO) [2,3,4,5]. While transient microglial activation is essential for tissue defense and repair, chronic or dysregulated reactivity may lead to synaptic dysfunction, persistent neuroinflammation, and neuronal injury. Indeed, excessive microglial activation has been implicated in the pathogenesis and progression of neurodegenerative diseases such as Alzheimer’s disease, Parkinson’s disease, and amyotrophic lateral sclerosis [6].

Emerging evidence suggests that mitochondrial metabolism is tightly linked to the functional state of microglia. These organelles are not only critical for cellular energy production via oxidative phosphorylation (OXPHOS) but also act as hubs for redox signaling, innate immune activation, and apoptotic regulation. Mitochondria-derived DAMPs (damage-associated molecular patterns), excessive ROS production, and shifts in mitochondrial dynamics are increasingly recognized as modulators of inflammatory responses in the CNS [7]. Notably, mitochondrial fragmentation—driven by enhanced fission and impaired fusion—has been associated with microglial proinflammatory activation and metabolic reprogramming [8].

In this context, mitochondrial dynamics, including fission (regulated by dynamin-related protein 1, Drp1) and fusion (mediated by mitofusins, Mfn1 and Mfn2), emerge as crucial determinants of microglial phenotype and bioenergetic plasticity [9]. Recent studies have shown that activated microglia can release dysfunctional mitochondria, which in turn promote neurotoxicity by inducing reactive astrocyte phenotypes [10]. Moreover, mitochondrial complex I activity in microglia has been shown to sustain chronic neuroinflammation via ROS generation, and its pharmacological inhibition reduces neuronal damage in animal models [11]. These findings point to mitochondria as both drivers and effectors of neuroimmune pathology.

Despite these advances, important questions remain regarding how inflammatory stimuli affect the integrity of mitochondrial structure, energy metabolism, and organelle quality control in microglia. Although previous studies have independently demonstrated that lipopolysaccharide (LPS) can induce both glycolytic [12,13] and mitochondrial dysfunction in microglia [8,14,15], the impact of this activation on metabolic flexibility—the ability of microglia to adapt their energy metabolism under stress—remains poorly understood.

To address this gap, we employed the BV-2 murine microglial cell line—a widely used model that retains key immune properties—to investigate how LPS, a prototypical Toll-like receptor 4 (TLR4) agonist, modulates mitochondrial function and metabolic adaptability in microglia. Our study integrates analyses of mitochondrial morphology, membrane potential, dynamics (fission/fusion proteins), mitophagy, glycolytic flux, and oxygen consumption to characterize the metabolic consequences of inflammatory stress in reactive microglia. We hypothesized that LPS-induced microglial activation triggers a cascade of mitochondrial dysfunction and reduced metabolic flexibility, which may contribute to persistent inflammatory phenotypes and bioenergetic failure. Understanding this cell-autonomous stress program may reveal novel targets for therapeutic intervention in neuroinflammatory and neurodegenerative disorders.

## 2. Results

### 2.1. LPS Induces Microglial Reactivity

**Figure 1:**
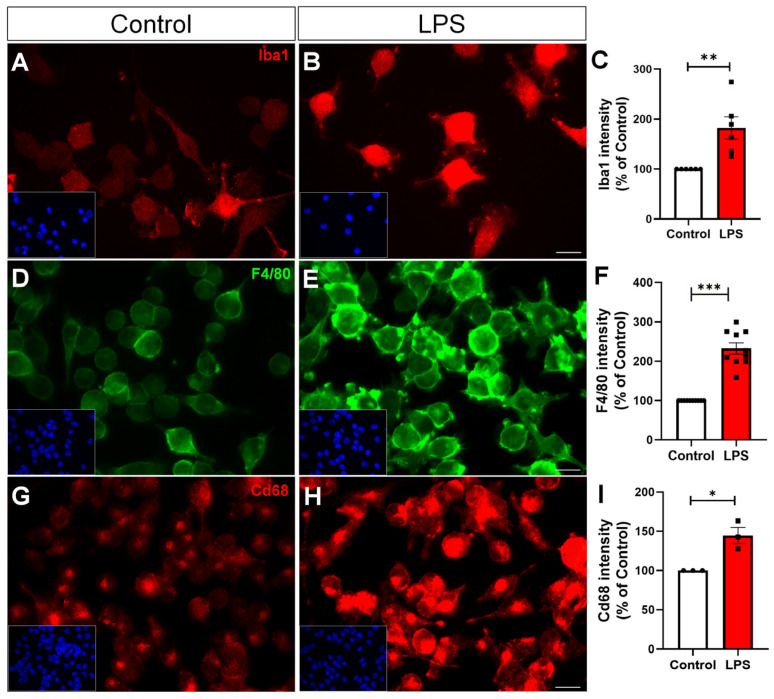
LPS induces microglial activation. Representative immunofluorescence images of BV-2 microglia treated with vehicle (control) or LPS (100 ng/mL, 24 h), showing increased expression of Iba1 (**A**–**C**), F4/80 (**D**–**F**), and Cd68 (**G**–**I**). Quantification indicates a significant increase in the levels of Iba1, F4/80, and Cd68 cells following LPS exposure, reflecting microglial reactivity. Individual data points represent independent cultures (*n* = 3–10 cultures/group). Statistical significance was determined using Student’s *t*-test. Data are expressed as mean ± SEM. * *p* < 0.05; ** *p* < 0.01; *** *p* < 0.001. Scale bars, 20 µm.

Microglial activation is a hallmark of neuroinflammation and is commonly assessed by the upregulation of specific markers such as IBA1, F4/80, and Cd68, which reflect cytoskeletal remodeling, immune activation, and phagocytic activity, respectively [16,17]. Murine BV-2 microglial cells were treated with 100 ng/mL of LPS for 24 h to induce an inflammatory response. Following treatment, immunocytochemistry revealed a significant increase in the expression of microglial activation markers. Iba1, a cytoplasmic protein involved in membrane ruffling and motility, showed an increase of 82% compared to the control (Figure 1A–C). F4/80, a glycoprotein expressed on the surface of activated murine macrophages, was elevated by 132% (Figure 1D–F). Cd68, a lysosomal-associated glycoprotein linked to phagocytic function, increased by 44% (Figure 1G–I), consistent with enhanced phagocytic activity. As expected, LPS treatment robustly promoted microglial activation, confirming the establishment of a reactive phenotype under inflammatory conditions.

### 2.2. LPS Increases H_2_O_2_, Nitric Oxide Production, Cytotoxicity, and Apoptosis in Microglia

**Figure 2:**
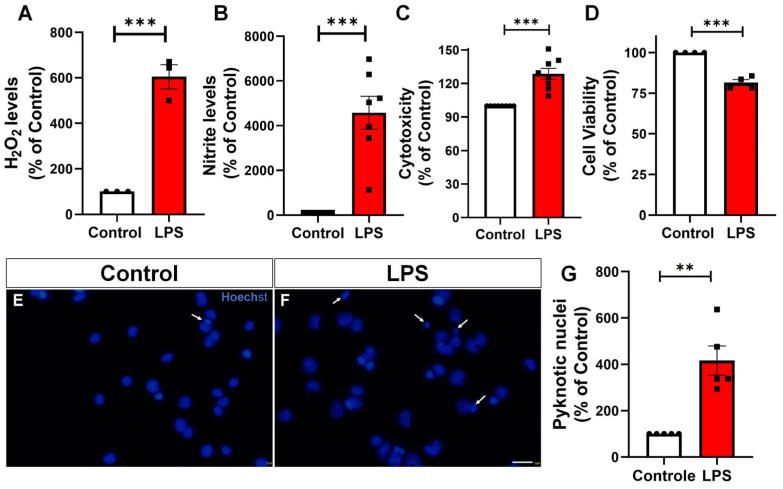
LPS promotes oxidative stress, nitrosative stress, cytotoxicity, apoptosis, and decreased metabolic viability in BV-2 microglia. Quantification of extracellular markers in culture supernatants of BV-2 microglia treated with vehicle or LPS (100 ng/mL, 24 h). (**A**) Hydrogen peroxide (H_2_O_2_) levels measured by Amplex Red assay. (**B**) Nitric oxide (NO) levels assessed via the Griess reaction. (**C**) LDH activity as a marker of cytotoxicity. (**D**) Mitochondrial activity and cell viability assessed by the MTT assay. (**E**,**F**) Representative fluorescence microscopy images of Hoechst stained nuclei showing control (**E**) and LPS-treated cells (**F**). Arrows indicate pyknotic nuclei. (**G**) Quantification of pyknotic nuclei revealed an approximately fourfold increase in apoptotic nuclear morphology in LPS-treated cells. Individual data points represent independent cultures (*n* = 3–9 cultures/group). Statistical significance was determined using Student’s *t*-test. Data are expressed as mean ± SEM. ** *p* < 0.010 and *** *p* < 0.001. Scale bar, 20 µm.

Activated microglia contribute to neuroinflammation by releasing ROS, nitric oxide, and inflammatory metabolites, which can exacerbate neuronal dysfunction and death [4,18]. To evaluate the functional consequences of LPS-induced activation, BV-2 microglia were treated with 100 ng/mL of LPS for 24 h. The culture supernatant was then analyzed for markers of oxidative stress and cytotoxicity. H_2_O_2_ production was significantly increased by sixfold compared to the control (Figure 2A). Nitrite (a stable form of nitric oxide) levels were elevated by 45-fold (Figure 2B). Lactate dehydrogenase (LDH) activity in the medium, an indicator of membrane damage and cytotoxicity, showed a rise of 40% (Figure 2C), reflecting LPS-induced cell stress. In addition, mitochondrial activity assessed by the MTT assay, revealed a significant reduction in cell viability following LPS treatment (Figure 2D), suggesting a potential impairment of mitochondrial function associated with the pro-inflammatory activation of microglia. To further evaluate cell death mechanisms, apoptosis was assessed through the analysis of pyknotic nuclei using Hoechst nuclear staining. LPS treatment led to an approximately fourfold increase in the number of pyknotic nuclei (Figure 2E–G), indicating enhanced apoptotic activity in microglial cells under inflammatory conditions. Collectively, these results indicate that LPS stimulation is associated with increased oxidative and nitrosative responses, as well as cytotoxicity and metabolic disturbances in microglial cells.

### 2.3. LPS Triggers Mitochondrial Membrane Potential Loss and Mitochondrial Fission in BV-2 Microglial Cells

**Figure 3:**
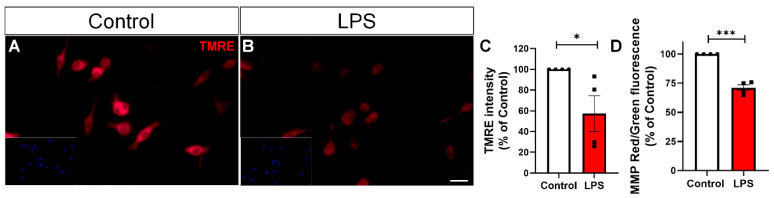
LPS induces mitochondrial membrane potential loss in BV-2 microglia. (**A**,**B**) Representative images of TMRE fluorescence in BV-2 microglia treated with vehicle or LPS (100 ng/mL, 24 h). (**C**) Quantification of TMRE fluorescence intensity as an indicator of mitochondrial membrane potential. (**D**) JC-1 assay, showing the ratio of red to green fluorescence as an additional measure of mitochondrial polarization status. Each data point represents an independent culture (*n* = 4 cultures per group). Statistical analysis was performed using Student’s *t*-test. Data are presented as mean ± SEM. * *p* < 0.05; *** *p* < 0.001. Scale bar, 20 µm.

**Figure 4:**
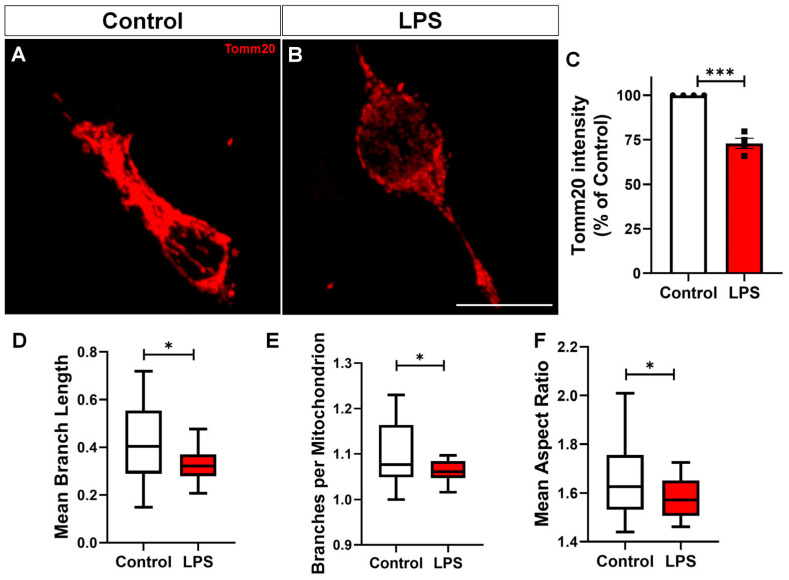
LPS induces mitochondrial fragmentation and reduces the Tomm20 signal in BV-2 microglia. (**A**,**B**) Representative immunofluorescence images of Tomm20-labeled mitochondria in BV-2 microglia treated with vehicle (Control) or LPS (100 ng/mL, 24 h). (**C**) Quantification of Tomm20 fluorescence intensity, indicating reduced mitochondrial content in LPS-treated cells. (**D**–**F**) Mitochondrial morphology analysis using the MiNA plugin in FIJI/ImageJ showing reduced (**D**) mean branch length, (**E**) number of branches per mitochondrion, and (**F**) aspect ratio in LPS-treated cells, consistent with increased mitochondrial fragmentation. Analyses in (**D**–**F**) were performed on four independent cultures, with six fields per culture (24 fields per experimental condition). Data are expressed as mean ± SEM. Statistical significance was determined using Student’s *t*-test. * *p* < 0.05, *** *p* < 0.001. Scale bar, 20 µm.

Mitochondria are central hubs for cellular metabolism and play a key role in regulating microglial activation [19]. To investigate whether LPS treatment alters mitochondrial function, we assessed mitochondrial membrane potential using two complementary approaches: JC-1 and TMRE staining, fluorescent dyes used to assess mitochondrial membrane potential. We observed a marked reduction in TMRE fluorescence intensity in LPS-treated BV-2 cultures (Figure 3A–C), indicating loss of membrane potential, which was corroborated by a reduction in the red/green fluorescence ratio of JC-1, confirming mitochondrial depolarization (Figure 3D). In addition, we examined mitochondrial morphology. Tomm20, a translocase of the outer mitochondrial membrane commonly used as a marker of mitochondrial mass and structure, showed significantly reduced immunoreactivity in the LPS-treated group, as revealed by quantification of integrated density (Figure 4A–C Morphometric analysis using the Mitochondrial Network Analysis (MiNA) tool revealed a significant decrease in mean mitochondrial branch length (Figure 4D), reflecting loss of mitochondrial elongation. The number of branches per mitochondrion (Figure 4E) was also reduced, suggesting diminished network complexity. Finally, the mean aspect ratio (Figure 4F), which reflects mitochondrial elongation, was lower in LPS-treated cells, confirming a shift toward a more rounded, fragmented mitochondrial phenotype.

### 2.4. Reactive Microglia Exhibit Altered Mitochondrial Dynamics and Impaired Mitophagy

**Figure 5:**
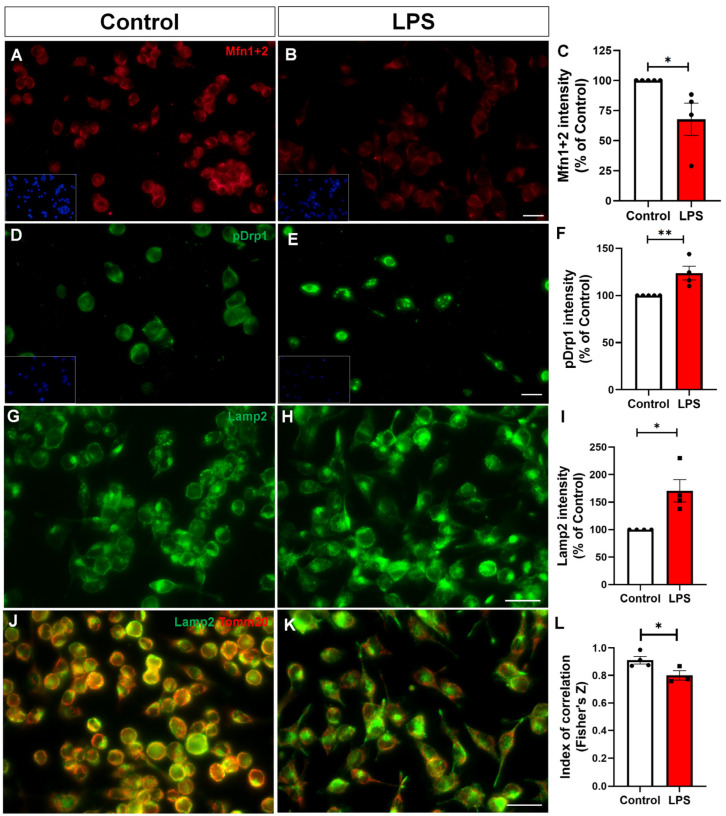
LPS modulates mitochondrial dynamics and impairs mitophagy in BV-2 microglia. (**A**,**B**) Representative immunofluorescence images of Mfn1/2 (red), key mitochondrial fusion proteins, in control and LPS-treated cells. (**C**) Quantification of Mfn1+2 fluorescence intensity. (**D**,**E**) Immunolabeling of phosphorylated Drp1 at serine 616 (p-Drp1), a key regulator of mitochondrial fission. (**F**) Quantification of p-Drp1 fluorescence intensity. (**G**,**H**) Images showing Lamp2 expression, a lysosomal marker. (**I**) Quantification of Lamp2 fluorescence intensity. (**J**,**K**) Representative merged images of Tomm20 (red) and Lamp2 (green) showing reduced colocalization upon LPS treatment. (**L**) Quantification of Tomm20–Lamp2 colocalization using Fisher’s Z-transformed Pearson’s correlation coefficient. Data represent mean ± SEM from independent cultures (*n* = 3–5 per group). Statistical analysis was performed using Student’s *t*-test. * *p* < 0.05, ** *p* < 0.01. Scale bars, 20 µm.

Furthermore, we evaluated the expression of proteins involved in mitochondrial dynamics. Immunofluorescence analysis revealed reduced labeling of mitofusins 1 and 2, GTPases located on the outer mitochondrial membrane that are essential for mitochondrial fusion, in LPS-treated cells (Figure 5A–C). Conversely, we observed increased levels of phosphorylated dynamin-related protein 1 at serine 616 (p-Drp1), a post-translational modification that promotes its recruitment to the outer mitochondrial membrane. p-Drp1 is a key regulator of mitochondrial fission, facilitating fragmentation upon activation (Figure 5D–F).

Mitophagy is a crucial autophagic process by which damaged or dysfunctional mitochondria are selectively identified, sequestered into autophagosomes, and subsequently degraded by lysosomes, playing a vital role in mitochondrial quality control and the maintenance of cellular homeostasis, particularly in metabolically active cells like microglia [20]. In this context, we investigated whether LPS treatment impairs mitophagy in BV-2 microglia. Following LPS exposure, BV-2 cells exhibited a significant increase in Lamp2 (lysosome-associated membrane protein 2) immunoreactivity, indicating lysosomal accumulation or activation (Figure 5G–I). Despite this upregulation, mitophagy was compromised, as evidenced by reduced colocalization between the mitochondrial marker Tomm20 and the lysosomal marker Lamp2 (Figure 5J–L). Together, these results indicate that reactive microglia exhibit a shift in mitochondrial dynamics toward excessive fission, accompanied by impaired mitophagy, ultimately contributing to mitochondrial dysfunction.

### 2.5. LPS Induces Mitochondrial Bioenergetic Shift in BV-2 Microglia

**Figure 6:**
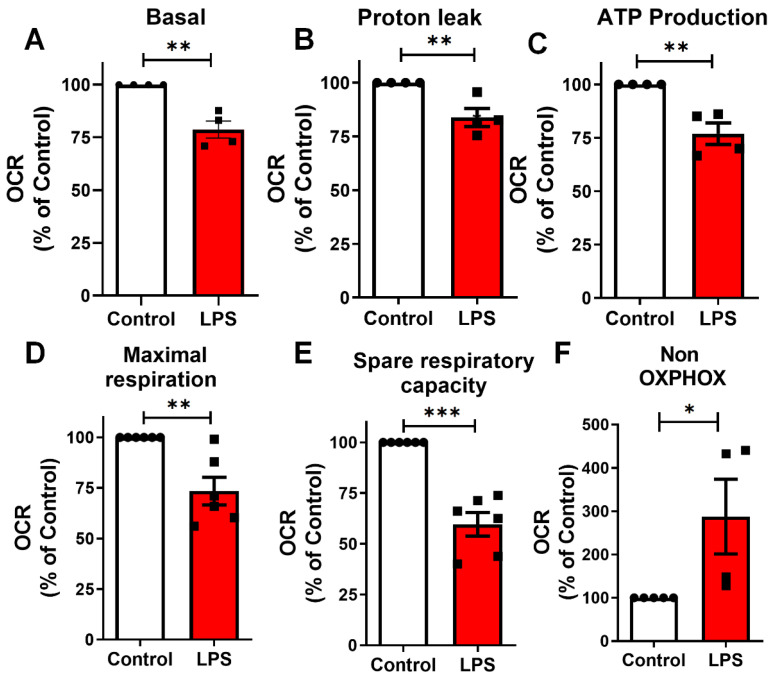
LPS impairs mitochondrial respiratory function in BV-2 microglia. High-resolution respirometry was performed to assess the oxygen consumption rate (OCR), an indicator of mitochondrial respiratory activity, in BV-2 microglia treated with LPS (100 ng/mL, 24 h) or vehicle. (**A**) Basal respiration, (**B**) proton leak, (**C**) ATP-linked respiration, (**D**) maximal respiration, (**E**) spare respiratory capacity, and (**F**) non-oxidative phosphorylation. Individual data points represent independent cultures (*n* = 3–5 cultures/group). Statistical significance was determined using Student’s *t*-test. Data are expressed as mean ± SEM. * *p* < 0.05; ** *p* < 0.01; *** *p* < 0.001.

Mitochondrial bioenergetics is essential for maintaining microglial homeostasis and immune responses, and its dysfunction has been linked to neuroinflammatory conditions [21]. To investigate the functional impact of LPS-induced mitochondrial alterations, high-resolution respirometry was performed using the OROBOROS O2k oxygraph system to directly assess mitochondrial respiratory function. BV-2 microglia treated with LPS (100 ng/mL, 24 h) exhibited a significant reduction in basal respiration, proton leak, ATP-linked respiration, maximal respiratory capacity, and spare respiratory capacity, respectively, when compared to control cells (Figure 6A–E). These findings indicate a broad impairment of mitochondrial respiratory function. Additionally, non-mitochondrial respiration was increased following LPS exposure (Figure 6F), suggesting a shift toward alternative oxygen-consuming pathways or increased background oxidative activity. Together, these results demonstrate that LPS induces profound mitochondrial bioenergetic alterations in reactive microglia.

### 2.6. LPS-Induced Alterations in Glycolytic Flux and Impaired ATP Maintenance in Microglia

**Figure 7:**
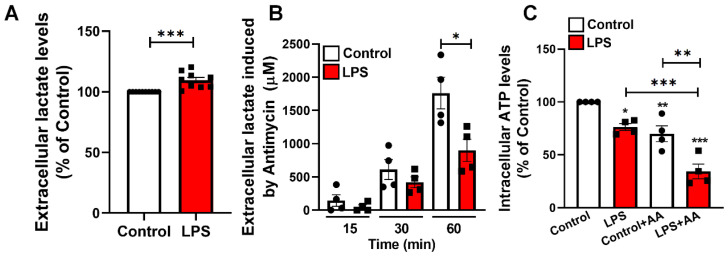
LPS impairs glycolytic flexibility and reduces ATP levels in BV-2 microglia. (**A**) Quantification of extracellular lactate after 24 h of LPS treatment. (**B**) Time-course measurement of extracellular lactate levels following the addition of 5 µM antimycin A (AA) at 15, 30, and 60 min. (**C**) Intracellular ATP levels basal and measured after 60 min of AA exposure in control and LPS-treated cells. Data are expressed as mean ± SEM. Statistical analysis was performed using Student’s *t*-test (**A**,**B**) and one-way ANOVA followed by Tukey’s post hoc test (**C**) * *p* < 0.05, ** *p* < 0.01, *** *p* < 0.001.

Given the morphological and functional alterations observed in microglia upon LPS exposure, we next investigated whether glycolytic metabolism was also affected. BV-2 microglial cultures treated with LPS (100 ng/mL, 24 h) exhibited a modest but significant increase in extracellular lactate levels compared to control conditions (Figure 7A), suggesting a shift toward glycolytic metabolism. To further explore glycolytic capacity, we assessed lactate release following the addition of antimycin A, a mitochondrial complex III inhibitor that forces cells to rely on glycolysis for ATP production. Time-course analysis of extracellular lactate showed that while both control and LPS-treated cultures increased lactate levels upon antimycin A exposure, LPS-treated cells displayed a blunted response at 60 min (Figure 7B), indicating a potential limitation in glycolytic compensation. Additionally, measurement of intracellular ATP levels revealed that LPS-treated cells exhibited reduced ATP content, which was further exacerbated by antimycin A treatment (Figure 7C). These findings suggest that LPS impairs not only mitochondrial respiration but also restricts the ability of microglia to sustain ATP levels through glycolysis under metabolic plasticity.

## 3. Discussion

Our study provides a detailed functional and morphological profile of LPS-induced reactivity in BV-2 microglial cells. In addition to the upregulation of classical activation markers (Iba1, F4/80, Cd68), LPS exposure triggered a multifaceted proinflammatory phenotype characterized by increased oxidative and nitrosative stress, cytotoxicity, evidence of apoptosis, and a metabolic shift toward glycolysis. We also observed pronounced mitochondrial alteration, including reduced membrane potential, decreased Tomm20 signal, fragmentation driven by altered expression of fusion (Mfn1/2) and fission (p-Drp1) proteins, and impaired energy production. These alterations culminated in reduced cell viability, underscoring the bioenergetic fragility of reactive microglia and highlighting key mechanisms that may contribute to neuroinflammatory damage.

Microglial activation is a hallmark of neuroinflammatory processes implicated in neurodegenerative diseases, stroke, and traumatic brain injury [22,23,24]. In our model, 24-h LPS stimulation led to a marked increase in Iba1, F4/80, and Cd68 expression, confirming the transition to a reactive, immune-competent phenotype [25,26]. These findings align with the known capacity of LPS, a potent TLR4 agonist, to activate innate immune pathways [27,28].

Functionally, LPS-activated BV-2 microglia displayed elevated production of reactive oxygen species (H_2_O_2_) and NO, key contributors to oxidative and nitrosative stress. This was accompanied by increased lactate dehydrogenase (LDH) release, indicating membrane damage, and a significant rise in extracellular lactate levels—hallmarks of metabolic reprogramming [29]. Although this shift may initially support the energy demands of immune activation, it reflects a departure from oxidative phosphorylation (OXPHOS), favoring a proinflammatory state [30]. Consistent with impaired mitochondrial function, MTT assay results revealed a significant reduction in cell viability, indicating decreased metabolic activity and reinforcing the notion that bioenergetic dysfunction is a key feature of LPS-induced microglial activation.

A central and novel contribution of our work lies in demonstrating that the reactive phenotype of LPS-stimulated microglia is closely associated with both structural and functional mitochondrial decline. LPS exposure led to a marked reduction in mitochondrial membrane potential, as demonstrated by decreased TMRE fluorescence and a lower red/green fluorescence ratio in the JC-1 assay. In parallel, Tomm20 immunostaining revealed diminished mitochondrial density, suggesting a loss of mitochondrial mass or structural integrity. The mitochondrial dysfunction observed in BV-2 cells likely represents a functional response to inflammatory stimulation rather than resulting from intrinsic or genetic mitochondrial defects, such as genetic mutations. Additionally, we observed a clear shift in mitochondrial dynamics toward fragmentation, supported by reduced expression of the fusion-related proteins Mfn1/2, and increased levels of p-Drp1, a key mediator of mitochondrial fission. Together, these findings highlight an imbalance in the fusion–fission machinery that favors mitochondrial fragmentation. This pattern is consistent with prior studies showing that altered mitochondrial morphology in activated microglia impairs calcium uptake and buffering, leading to intracellular calcium dyshomeostasis and disrupted signaling pathways—further reinforcing the pathophysiological relevance of mitochondrial dynamics in neuroinflammation [8,14]. It is important to note that mitochondrial fragmentation, as observed in our model, may not exclusively reflect shifts in mitochondrial dynamics but could also represent an early feature of programmed cell death. To address this possibility, we performed an additional morphological analysis and observed a significant increase in cells displaying pyknotic nuclei following LPS stimulation. These findings suggest that a proportion of the fragmented mitochondrial phenotype may be associated with apoptotic commitment. While our data support altered mitochondrial dynamics via Drp1 phosphorylation and Mfn1/2 downregulation, these processes may intersect with early apoptotic events. Similarly, age-related astrocyte dysfunction has been associated with increased mitochondrial fragmentation, which contributes to impaired metabolic support and reduced neuroprotective capacity in the aging brain [31,32].

Mitophagy is the selective autophagic removal of damaged or dysfunctional mitochondria [33]. Activation of mitophagy has been shown to restore mitochondrial function and reduce the proinflammatory response in BV-2 microglial cells exposed to LPS, also decreasing NLRP3 inflammasome activation both in vitro and in vivo [34]. Despite the increased expression of the lysosomal marker Lamp2, mitophagy was impaired, as shown by reduced colocalization of mitochondria with lysosomes. This suggests a failure in mitochondrial quality control [35]. Possibly reflecting multiple contributing factors, including a blockade in the autophagic flux under inflammatory stress [36]), inefficient clearance of damaged mitochondria leading to their accumulation and exacerbation of cellular stress [37], lysosomal overload or dysfunction when the demand for organelle degradation exceeds the cell’s degradative capacity [38], and alterations in the actin cytoskeleton—particularly involving the Arp2/3 complex and cofilin—that may impair the trafficking and fusion of mitophagosomes with lysosomes [39], future studies are warranted to dissect the relative contribution of these mechanisms and to determine whether targeting cytoskeletal dynamics or enhancing lysosomal function can restore mitophagy and mitochondrial homeostasis in chronically activated microglia.

These structural abnormalities were paralleled by a broad suppression of mitochondrial respiration. High-resolution respirometry revealed significant deficits in basal respiration, ATP-linked respiration, maximal respiratory capacity, and spare respiratory capacity. Together, these changes highlight a collapse in mitochondrial energy production and reinforce the hypothesis of a cell-autonomous metabolic dysfunction in reactive microglia [40].

The increased non-mitochondrial oxygen consumption in LPS-treated microglia may result from the activation of NOX2 and COX-2, which are known to consume oxygen and generate ROS [41]. NOX2 activity leads to elevated H_2_O_2_ production, contributing to oxidative stress and cell death, as possibly observed in our study.

Previous studies have demonstrated that microglial activation upregulates glycolytic enzymes, indicating a metabolic reprogramming toward glycolysis [13,42,43]. This is consistent with our findings, in which LPS-treated BV-2 microglial cells exhibited elevated levels of extracellular lactate. This increase may stem from enhanced glycolytic flux to compensate for mitochondrial dysfunction or, alternatively, may reflect an adaptive response to limit inflammatory damage—particularly given that L-lactate has been shown to suppress LPS-induced inflammation in microglia [44]. Upon mitochondrial inhibition with antimycin A, LPS-primed BV-2 cells exhibited a more pronounced reduction in intracellular ATP levels compared to control cells, suggesting that prior LPS activation impairs metabolic flexibility and limits the capacity of microglia to maintain energy homeostasis under mitochondrial stress. This indicates that reactive microglia may exhibit impaired metabolic plasticity, potentially due to preexisting glycolytic engagement or underlying mitochondrial dysfunction, ultimately contributing to exacerbated ATP depletion upon respiratory chain inhibition. The observed interplay between impaired OXPHOS, metabolic shift toward glycolysis, and disrupted mitophagy suggests the presence of a self-perpetuating cycle of bioenergetic stress, sustained inflammation, and organelle dysfunction [45,46].

A key limitation of this study lies in the use of the BV-2 microglial cell line. While BV-2 cells offer practical advantages and are widely employed in neuroinflammation research, they do not fully recapitulate the phenotype, transcriptomic landscape, or functional adaptability of primary microglia—particularly those of human origin [47,48]. As an immortalized murine cell line, BV-2 cells exhibit a degree of basal activation and may differ in their responsiveness to stimuli when compared to microglia in ex vivo or in vivo conditions. Nevertheless, BV-2 cells remain a valuable and extensively validated model for dissecting microglial mechanisms and conducting high-throughput functional screening.

Our findings support the emerging concept that mitochondrial integrity is central to microglial homeostasis and that proinflammatory stimuli, such as LPS, can trigger a self-sustaining program of mitochondrial and metabolic failure. More recently, it has been shown that mitochondrial toxins, which are potent mitochondrial inhibitors, can directly activate microglia via the p38 MAPK pathway, further supporting the notion that mitochondrial dysfunction plays a direct role in driving microglial reactivity [49] or inducing cell death through apoptosis [50]. The resulting accumulation of mitochondrial DAMPs, loss of respiratory flexibility, and increased glycolytic byproducts may amplify neuroinflammation and contribute to neurodegenerative progression [51].

Moreover, elucidating the molecular pathways that connect LPS signaling to mitochondrial fragmentation and impaired mitophagy—such as those involving Drp1, the Pink1/Parkin axis, may reveal novel therapeutic targets to restore mitochondrial integrity and metabolic resilience in reactive microglia.

## 4. Conclusions

This study provides an integrated characterization of LPS-induced microglial activation, revealing profound alterations in mitochondrial structure, function, and metabolic homeostasis. LPS-treated BV-2 microglia exhibited increased mitochondrial fragmentation, reduced membrane potential, and impaired respiratory function, accompanied by signs of defective mitophagy. Additionally, we observed elevated basal extracellular lactate levels and reduced MTT-based viability, indicating a metabolic shift toward glycolysis. Upon mitochondrial inhibition with antimycin A, LPS-primed cells showed a diminished glycolytic response and exacerbated ATP depletion, reflecting impaired metabolic flexibility. These findings suggest that mitochondrial dysfunction, altered metabolic plasticity, and disrupted energy metabolism are central features of the reactive microglial phenotype. Targeting mitochondrial dynamics and mitophagy may represent promising therapeutic strategies to modulate microglial reactivity in neuroinflammatory and neurodegenerative diseases.

## 5. Materials and Methods

### 5.1. Cell Culture

The murine microglial cell line BV-2 was obtained from the Rio de Janeiro Cell Bank (Banco de Células do Rio de Janeiro - BCRJ, Rio de Janeiro, Brazil, reference #0356). Cells were cultured in Dulbecco’s Modified Eagle Medium (DMEM) supplemented with Ham’s F-12 nutrient mixture (Thermo Fisher Scientific, Waltham, MA, USA, Cat. 12400024), 2 mM L-glutamine, 100 U/mL penicillin, 100 µg/mL streptomycin, 0.25 µg/mL amphotericin B (Fungizone), and 10% (*v*/*v*) fetal bovine serum (FBS; Thermo Fisher Scientific, Cat. 12657029). Cultures were maintained at 37 °C in a humidified atmosphere containing 5% CO_2_. Cells were passaged upon reaching 70–80% confluence. For experimental procedures, BV-2 cells were seeded onto 24-well plates containing glass coverslips at a density of 50,000 cells per well or into 60 mm culture dishes at a density of 1 × 10^6^ cells per dish. Cells were allowed to adhere under standard culture conditions. Routine screening for mycoplasma contamination was performed by evaluating nuclear morphology under DAPI staining at 100× magnification. No evidence of mycoplasma contamination was observed throughout the experimental period.

### 5.2. Treatment

BV-2 cells were maintained in serum-free medium for 4 h prior to stimulation. LPS (Sigma-Aldrich, St. Louis, MO, USA, Cat. L6143) was then added at a final concentration of 100 ng/mL, and the cells were incubated for an additional 24 h. This concentration was selected based on previous studies demonstrating its efficacy in inducing a robust and sustained inflammatory response in BV-2 microglial cells without causing overt cytotoxicity [14]. Phosphate-buffered saline (PBS) was used as the vehicle control in all experiments. Following LPS treatment, BV-2 cells were subjected to morphological and functional analyses. For the quantification of extracellular metabolites (lactate, LDH, H_2_O_2_, and nitrite), 24 h after treatment, the conditioned medium was collected and centrifuged at 1500× *g* for 10 min. The resulting clarified supernatant was then used for subsequent biochemical analyses.

### 5.3. Immunocytochemistry

BV-2 cell cultures were fixed with 4% paraformaldehyde in phosphate-buffered saline (PBS) at pH 7.4 for 15 min. To block non-specific binding sites, the cultures were then incubated with a solution of 3% bovine serum albumin, 5% normal goat serum, and 0.2% Triton X-100 in PBS for 1 h at room temperature. Following blocking, the cells were incubated overnight with primary antibodies: Rabbit anti-Cd68 (1:300; Abcam, Cambridge, UK, Cat. 125212); Rat anti-F4/80 (1:300; Bio-Rad, Hercules, CA, USA, Cat. #163156); Rabbit anti-Iba1 (1:1000; Fujifilm Wako, Osaka, Japan, Cat. 019-19741); Rat anti-Lamp2 (1:300; Merck Millipore, Darmstadt, Germany Cat. #MABC40); Rabbit anti-Tomm20 (1:1000; Abcam, Cambridge, UK, Cat. ab186735); mouse anti-Mitofusin1 + Mitofusin2 (1:300 dilution; Abcam, Cambridge, UK, Cat. ab57602) and rabbit phospho-Drp1 (Ser616) (1:100, Thermo Fisher Scientific, Waltham, MA, USA, Cat. PA5-64821). After thorough washing with PBS, secondary antibodies (Alexa Fluor 555 or 488-conjugated goat anti-rabbit/mouse IgG; Thermo Fisher Scientific, Waltham, MA, USA) were applied at appropriate dilutions (1:1000 Alexa Fluor 555 or 1:300 Alexa Fluor 488) for 2 h at room temperature. Finally, nuclei were counterstained with DAPI (Sigma-Aldrich) and the cells were observed using a Leica SPE confocal microscope (Leica Microsystems, Wetzlar, Germany), a Nikon TE2000 microscope (Nikon Instruments Inc., Tokyo, Japan) or a Nexcope NIB-620FL microscope (Ningbo Yongxin Optics Co., Ningbo, China).

### 5.4. Immunofluorescence Analysis

Densitometry for the immunocytochemistry images was performed using integrated density values generated with the ImageJ program version 1.53t (National Institutes of Health, USA, RRID:SCR_003070). Immunocytochemistry data were collected from at least 10 fields per coverslip, in duplicate. The integrated density value was divided by the number of cells in each field. Mitochondrial morphology was analyzed using the Mitochondrial Network Analysis (MiNA) toolset in FIJI/ImageJ [52]. Fluorescence images of cells labeled with Tomm20 were preprocessed using contrast enhancement and noise reduction. MiNA generated skeletonized mitochondrial networks, from which morphological parameters were extracted. All analyses were performed using identical settings across experimental groups. Tomm20 colocalization with Lamp2 was quantified using the Colocalization Colormap plugin in ImageJ (National Institutes of Health, Bethesda, MD, USA). This plugin computes the normalized mean deviation product (nMDP), which reflects the correlation between pixel intensities [53]. For statistical comparison, the resulting correlation indices were transformed using Fisher’s Z transformation.

Pyknotic nuclei were defined as condensed, highly fluorescent Hoechst-stained nuclei with markedly reduced nuclear area and a rounded shape, clearly distinct from the larger, diffuse, and homogeneous nuclear morphology observed in healthy cells [54]. The percentage of cells with pyknotic nuclei was calculated as the number of pyknotic nuclei divided by the total number of Hoechst-positive nuclei in each field. Data were normalized to the control group, which was set at 100%, to facilitate comparison across experimental conditions.

### 5.5. Measurement of Extracellular Hydrogen Peroxide

Extracellular hydrogen peroxide (H_2_O_2_) levels were quantified using the Amplex™ Red Hydrogen Peroxide/Peroxidase Assay Kit (Invitrogen, Thermo Fisher Scientific, Cat. A22188), following the manufacturer’s instructions with minor modifications. After treatment, 50 µL of culture supernatant was collected and incubated with 50 µL of reaction buffer containing 0.1 mM Amplex Red reagent and 0.2 U/mL horseradish peroxidase in 1× reaction buffer (provided with the kit). The mixture was incubated for 30 min at room temperature in the dark. Fluorescence was measured using a microplate reader (GloMax Microplate Reader, Promega Corporation, Madison, WI, USA) with excitation at 520 nm and emission at 580–640 nm. Results were normalized to cell number, as appropriate, and expressed as a percentage of control.

### 5.6. Cell Viability Assessment

We determined cellular cytotoxicity by measuring the extracellular activity of lactate dehydrogenase (LDH), which is an indicator of cellular injury. The LDH activity assay was performed using 50 μL of culture medium following the manufacturer’s instructions (CytoTox-Glo™ Cytotoxicity Assay, Promega Corporation, Madison, WI, USA, Cat. G1780).

### 5.7. Extracellular Lactate Levels

Extracellular lactate levels in the culture medium were assessed using a commercial kit (Labtest Diagnóstica S/A, Lagoa Santa, MG, Brazil, Cat. 138). A total of 2 µL of the medium was used for 100 µL of the reaction mix. To test the contribution of the glycolytic pathway to lactate production, cultures were washed with Gey’s buffer (NaCl: 137 mM; CaCl_2_: 1.53 mM; KCl: 4.96 mM; MgCl_2_: 1.03 mM; KH_2_PO_4_: 0.22 mM; Na_2_HPO_4_: 0.85 mM; MgSO_4_: 0.28 mM; NaHCO_3_: 2.70 mM; and C_6_H_12_O_6_: 41.64 mM), followed by the addition of medium containing 5 µM of antimycin to inhibit mitochondrial function and activate the glycolytic pathway. Culture medium was collected at 15, 30, and 60 min, and lactate levels were assessed at these time points. Phase-contrast microscopy performed immediately after treatment showed no evidence of overt cytotoxicity or morphological alterations.

### 5.8. Nitrite Levels

Nitrite (NO_2_^−^) quantification was performed as previously described [55,56]. Briefly, 50 μL of culture medium was mixed with 50 μL of 1% sulfanilamide prepared in 10% phosphoric acid (H_3_PO_4_) and incubated for 5 min at room temperature. Then, 50 μL of 0.1% N-(1-naphthyl)ethylenediamine prepared in water was added, followed by an additional 5-min incubation. Absorbance was subsequently measured at 540 nm.

### 5.9. Intracellular ATP Levels

Intracellular ATP quantification was performed using the CellTiter-Glo^®^ 2.0 luminescent assay (Promega Corporation, Madison, WI, USA, Cat. #G7572), following the manufacturer’s instructions. BV-2 cells were seeded in 24-well plates at a previously optimized density and treated with LPS (100 ng/mL) for 24 h. After treatment, cells were washed and incubated in fresh medium. ATP levels were measured under two distinct conditions: (i) immediately after a gentle wash with Gey’s balanced salt solution (basal condition, without antimycin A), and (ii) 60 min after the addition of 5 µM antimycin A, a mitochondrial complex III inhibitor used to impose respiratory chain stress. At each time point, CellTiter-Glo^®^ 2.0 reagent was added directly to the wells. Plates were shaken for 10 min on an orbital shaker to promote cell lysis, followed by a 10-min incubation at room temperature for signal stabilization. Lysates were transferred to white opaque 96-well plates, and luminescence was recorded using a GloMax^®^ plate reader (Promega) with an integration time of 0.5 s per well. Luminescence values were normalized to the control group.

### 5.10. Assessment of Mitochondrial Membrane Potential Using TMRE and JC-1 Dyes

For the assessment of mitochondrial membrane potential, BV-2 cells were stained with TMRE (Thermo Fisher Scientific, Cat. #T669). The culture medium was removed, and cells were incubated with 100 nM TMRE diluted in phenol red–free DMEM/F12 for 30 min at 37 °C in the dark. After incubation, cells were washed twice with Gey’s balanced salt solution and maintained in phenol red–free DMEM/F12 for imaging. Fluorescence images were acquired using a fluorescence microscope. Mitochondrial membrane potential was quantified by comparing TMRE fluorescence intensity between control and LPS-treated cultures. For each condition, five random fields per well were captured. TMRE fluorescence intensity was normalized to the number of nuclei per field, determined by Hoechst 33,342 staining, to account for variations in cell density.

Mitochondrial membrane potential was also assessed using the JC-1 dye (Thermo Fisher Scientific, Cat. #T3168). BV-2 cells were seeded exclusively in 96-well plates at a density of 20,000 cells per well, allowed to adhere for 1 h, and then treated with LPS (100 ng/mL) for 24 h. After treatment, JC-1 was added to each well at a final concentration of 2 µg/mL, and the plate was incubated for 30 min at 37 °C in the dark. Following incubation, cells were washed twice with Gey’s balanced salt solution and maintained in phenol red–free DMEM/F12 during fluorescence acquisition. Readings were performed using a GloMax^®^ Discover plate reader (Promega). Red fluorescence (J-aggregates, indicative of polarized mitochondria) was measured at Ex/Em 520/580–640 nm, and green fluorescence (monomeric form, indicative of depolarized mitochondria) at Ex/Em 475/500–550 nm. Mitochondrial membrane potential was expressed as the red/green fluorescence ratio, normalized to the untreated control.

### 5.11. Mitochondrial Respiration in Microglial Cultures

Murine microglial BV-2 cells were cultured and treated with LPS according to the experimental protocol previously described by our group [57,58,59]. After 24 h of treatment, cells were adjusted to 1×10^6^ cells per run for analysis in an Oroboros O2k respirometer (Oroboros Instruments, Innsbruck, Austria). Oxygen consumption rates (OCR) were monitored in real-time using Oroboros DatLab software. Initially, basal respiration was recorded for 5 min. Subsequently, 0.5 µg/mL oligomycin (ATP synthase inhibitor), 1 µM FCCP (uncoupler), and 4 µM antimycin A (Complex III inhibitor) were sequentially added, with OCR recorded for at least 3 min after each addition. The following mitochondrial respiration parameters were determined: Non-mitochondrial respiration, calculated as the OCR after antimycin A addition; basal respiration, defined as OCR before additions minus non-mitochondrial respiration; ATP-linked respiration, representing basal OCR minus OCR after oligomycin addition; maximal respiration, calculated as OCR after FCCP addition minus non-mitochondrial respiration; spare respiratory capacity, determined as maximal respiration minus basal respiration; and proton leak, defined as OCR after oligomycin minus non-mitochondrial respiration.

### 5.12. MTT Assay for Cell Viability

Cell viability was assessed using the MTT assay (3-[4,5-dimethylthiazol-2-yl]-2,5-diphenyltetrazolium bromide; Sigma-Aldrich, Cat. #M5655). BV-2 cells were seeded in 96-well plates at a density of 20,000 cells per well and allowed to adhere for 1 h. Cells were then treated with LPS (100 ng/mL) for 24 h. After treatment, 10 µL of MTT solution (5 mg/mL in PBS) was added to each well containing 100 µL of culture medium, and the plate was incubated for 2 h at 37 °C in the dark. Following incubation, the medium was carefully removed, and 100 µL of DMSO was added to each well to dissolve the formazan crystals. The plate was agitated for 5 min at room temperature to ensure complete solubilization. Absorbance was measured at 560 nm using a microplate reader (GloMax^®^, Promega). Viability was expressed as a percentage relative to the untreated control group. All conditions were tested in technical triplicate and at least three independent experiments.

### 5.13. Data and Statistical Analysis

Statistical analysis of quantitative data was performed using GraphPad Prism software version 8.0 (GraphPad Software, La Jolla, CA, USA). A 95% confidence interval was applied, and p-values less than 0.05 were considered statistically significant. Results are presented as mean ± SEM, except for the control group when specified otherwise. For comparisons between two groups, the Student’s *t*-test was used. For comparisons involving more than two groups or multiple conditions, one-way ANOVA followed by Tukey’s multiple comparisons post hoc test was applied. Detailed information on statistical tests, p-values, and sample sizes is provided in the respective figure legends.

## Data Availability

The original contributions presented in this study are included in the article. Further inquiries can be directed to the corresponding author(s).

## Abbreviations

Cd68	Cluster of differentiation 68
CNS	Central nervous system
COX-2	Cyclooxygenase-2
DAMPs	Danger-associated molecular patterns
DMEM	Dulbecco’s Modified Eagle Medium
H_2_O_2_	Hydrogen peroxide
IBA1	Ionized calcium-binding adaptor molecule 1
JC-1	5,5′,6,6′-tetrachloro-1,1′,3,3′-tetraethylbenzimidazolylcarbocyanine iodide
Lamp2	Lysosome-associated membrane protein 2
LDH	Lactate dehydrogenase
LPS	Lipopolysaccharide
Mfn1	Mitofusin 1
Mfn2	Mitofusin 2
MiNA	Mitochondrial Network Analysis
MTT	3-(4,5-dimethylthiazol-2-yl)-2,5-diphenyltetrazolium bromide
NO	Nitric oxide
NOX2	NADPH oxidase 2
OXPHOS	Oxidative phosphorylation
p-DRP	Phosphorylated dynamin-related protein 1 at serine 616
ROS	Reactive oxygen species
TLR4	Toll-like receptor 4
TMRE	Tetramethylrhodamine ethyl ester
Tomm20	Translocase of outer mitochondrial membrane 20
